# Progressive early passive and active exercise therapy after surgical rotator cuff repair – study protocol for a randomized controlled trial (the CUT-N-MOVE trial)

**DOI:** 10.1186/s13063-018-2839-5

**Published:** 2018-09-03

**Authors:** Birgitte Hougs Kjær, S. Peter Magnusson, Susan Warming, Marius Henriksen, Michael Rindom Krogsgaard, Birgit Juul-Kristensen

**Affiliations:** 1 Department of Physical and Occupational Therapy, Bispebjerg-Frederiksberg Hospital, Bispebjerg Bakke 23, DK-2400 Copenhagen, Denmark; 2 Department of Sports Science and Clinical Biomechanics, University of Southern Denmark, Campusvej 55, DK-5230 Odense, Denmark; 3 Institute of Sports Medicine, Department of Orthopaedic Surgery M, Copenhagen Bispebjerg-Frederiksberg Hospital, and Center for Healthy Aging, Faculty of Health and Medical Sciences, University of Copenhagen, Copenhagen, Denmark; 4 Section for Sports Traumatology M51, Department of Orthopaedic Surgery, Bispebjerg-Frederiksberg Hospital, Bispebjerg Bakke 23, DK-2400 Copenhagen, Denmark; 5 The Parker Institute, Bispebjerg-Frederiksberg Hospital, Ndr. Fasanvej 57, DK-2000 Frederiksberg, Copenhagen Denmark

**Keywords:** Shoulder, Rotator cuff, Tear, Rupture, Physiotherapy, Rehabilitation, Exercise therapy

## Abstract

**Background:**

Rotator cuff tear is a common cause of shoulder disability and results in patients predominantly complaining of pain and loss of motion and strength. Traumatic rotator cuff tears are typically managed surgically followed by ~ 20 weeks of rehabilitation. However, the timing and intensity of the postoperative rehabilitation strategy required to reach an optimal clinical outcome is unknown. Early controlled and gradually increased tendon loading has been suggested to positively influence tendon healing and recovery. The aim of this trial is therefore to examine the effect of a progressive rehabilitation strategy on pain, physical function and quality of life compared to usual care (that limits tendon loading in the early postoperative phase) in patients who have a rotator cuff repair of a traumatic tear.

**Methods:**

The current study is a randomized, controlled, outcome-assessor blinded, multicenter, superiority trial with a two-group paralleled design. A total of 100 patients with surgically repaired traumatic rotator cuff tears will be recruited from up to three orthopedic departments in Denmark, and randomized to either a progressive early passive and active movement program or a limited early passive movement program (usual care). The primary outcome measure will be the change from pre-surgery to 12 weeks post-surgery in the Western Ontario Rotator Cuff Index questionnaire. Secondary outcomes include the Disabilities Arm, Shoulder and Hand questionnaire (DASH), range of motion, strength and tendon healing characteristics from ultrasound measurements at 12 months follow up.

**Discussion:**

We hypothesized that patients who receive the progressive rehabilitation strategy will benefit more with respect to pain reduction, physical function and quality of life than those who receive care as usual. If this is confirmed our study can be used clinically to enhance the recovery of patients with traumatic rotator cuff tear.

**Trial registration:**

ClinicalTrials.gov, NCT02969135. Registered on 15 November 2016.

**Electronic supplementary material:**

The online version of this article (10.1186/s13063-018-2839-5) contains supplementary material, which is available to authorized users.

## Background

Shoulder pain is the third most common musculoskeletal disorder with a lifetime prevalence of 30% [1, 2]. Shoulder disorders are often persistent and recurrent, and 54% of patients report some symptoms after 3 years [1, 3]. Moreover, it often impacts people’s daily life and work capacity dramatically [4–6]. Rotator cuff tears, which can be due to trauma or degeneration [7], are common causes of chronic shoulder pain and disability, especially with advancing age [1, 3, 8, 9]. The predominant shoulder complaints among patients are pain, loss of motion and strength during arm elevation, which results in loss of function [3, 9]. One of the criteria for surgery is symptomatic full-thickness tears, or partial-thickness tears that are symptomatically resistant to non-surgical intervention [10, 11]. The incidence of rotator cuff surgery, based on surgery for both non-traumatic and traumatic tears, is increasing worldwide [12, 13], and The National Patient Register in Denmark recorded 730 rotator cuff repairs in 2006 and 990 in 2012, which represents a 35% increase [14].

The Danish National Clinical Guideline on selected shoulder disorders recommends that these patients are offered rehabilitation after surgical repair, and that the shoulder is immobilized post-surgery (usual care) [14]. However, the existing clinical studies of postoperative treatment targeting patients with rotator cuff repair are of moderate to low quality (lack of blinding, small sample sizes, lack/poor responsiveness on outcomes, and/or with only per protocol analyses) [15–20], making it difficult to unequivocally conclude what treatment regimen is the best. Studies on the timing of rehabilitation have showed a smaller positive short-term effect of early active rehabilitation (from postoperative day 21) compared to later rehabilitation [15], but no superior effect of early passive exercises [17, 19, 20]. A small or no effect was seen in studies focusing on the intensity of rehabilitation, including passive exercises versus immobilization [16] and progressive versus limited early passive exercises [18]. Recent systematic reviews on the effect of rehabilitation after rotator cuff surgery confirms that early range of motion (ROM) exercises accelerate healing, reduce stiffness, do not increase the risk of re-tear and that immobilization does not increase tendon healing or the clinical outcome [21–24]. However, high-quality and adequately powered trials with standardized treatment protocols testing early initiation of rehabilitation and gradual introduction of functional load and including important clinical outcomes such as return to work are needed [25, 26].

Research on rotator cuff tears often focuses on improvement of surgery techniques [9, 27–31], biomechanics and biology of tendon healing [16, 32, 33]; however, little is known about the effects of postoperative rehabilitation. Human autopsy studies indicate that the tendon can regain its ability to transmit almost the same amount of force as an intact tendon if it is fully repaired [10, 33]. The magnitude of retraction will markedly influence the rotator cuff muscle generating capacity [33]. Based on autopsy and physiological data [34] it has been suggested that rehabilitation should include reestablishment of joint ROM, shoulder function and muscular strength while considering the healing time of the repaired tendon.

The metabolic turnover of tendon tissue is slower than in muscle [35–37], and therefore a controlled and gradual increase in tendon loading has been suggested to achieve optimal tendon healing [38, 39]. This includes starting rehabilitation in the proliferative phase of healing and continuing into the remodeling phase so that the collagen is subjected to a load that is beneficial for the formation and the final strength of the tendon [35]. This means that postoperative immobilization may decrease tendon strength [40–42], and therefore increase the risk of a re-tear [34, 43, 44]. Further, immobilization may also result in adhesions and decreased ROM [38, 45]. Consequently, early passive motion postoperatively has gained acceptance [35, 38, 46].

In summary, no high-quality study has evaluated the combined effect of early (= the time of initiation) and progressive (= with an increased intensity, also including active muscle contraction) postoperative exercises on physical function, pain, quality of life and biological tendon healing. Therefore, the short-term (12 weeks) and long-term (12 months) effects of an early and targeted progressive postoperative exercise therapy program remain to be investigated. The primary aim of this trial is to evaluate the effect of a 12-week progressive rehabilitation (PR) strategy on shoulder function compared to usual care (UC) in patients recovering from surgical treatment of rotator cuff tears. The secondary aims include the effects on shoulder ROM, muscle strength, return to work rates and tendon healing characteristics. Finally, a prediction of health outcomes will be performed in both groups.

## Methods/design

**Fig. 1:**
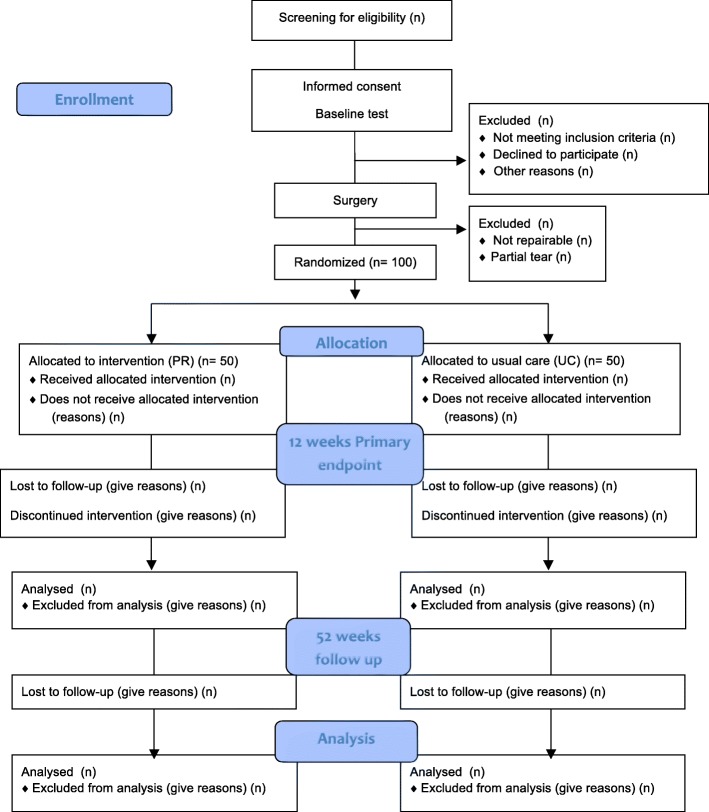
Expected flow of patients through the study. PR, progressive rehabilitation; UC, usual care

This is a randomized, controlled, outcome-assessor blinded, superiority trial, called CUT-N-MOVE with a two-group parallel design comparing a progressive rehabilitation (PR) strategy with usual care (UC). It is a multicenter study with two phases; the first phase includes the main trial with a baseline and a 12-week follow-up, which corresponds to the planned duration of the experimental individualized rehabilitation program (Fig. 1). The primary endpoint is 12 weeks from the baseline.

The second phase includes the secondary endpoint 52 weeks after baseline, at which point Ultrasound (US) measurements are included in the outcome assessments. During the 40-week follow up period (from week 12 to week 52) most patients continue standard rehabilitation in the community for 4–8 weeks. The community care providers are not informed about initial treatment to which each patient has been allocated (unless the patients tell them) and can thus be considered quasi-blinded.

After 12 weeks no study-related activities are performed, except for the 12-month follow up and treatment of any adverse events that might occur during the trial (52 weeks). Patients are randomized equally (1:1) to receive either PR or UC (Fig. 1).

### Patients and settings

Patients are recruited from the Section for Sports Traumatology, Department of Orthopedic Surgery, Bispebjerg-Frederiksberg Hospital and from The Shoulder-Elbow Unit, Herlev-Gentofte Hospital, both in Denmark. Additional inclusion sites may be added.

Patients may be enrolled in the study provided they meet all of the inclusion criteria:

- Age minimum 18 years
- A clinical diagnosis of traumatic full-thickness rotator cuff (RC) tear involving the supraspinatus tendon (full thickness and width) verified by arthroscopy
- A repairable tear

In trauma the trauma mechanism should be described. Typically it will be a forced abduction and external rotation to mitigate for a fall, a fall on the outstretched arm, a pull in the arm or a shoulder luxation. No previous symptoms are implied within the definition of a traumatic tear.

Patients are excluded if they fulfill any of the following exclusion criteria:

1. A non-traumatic RC tear of the shoulder
2. An isolated teres minor or subscapularis tear
3. A partial-thickness/partial-width tear
4. Prior shoulder surgery (all shoulder joints)
5. A clinical diagnosis of glenohumeral osteoarthritis (OA), rheumatoid arthritis or periarthrosis
6. Unable to speak or read Danish
7. Unable to perform and maintain the exercise therapy
8. Other conditions that negatively influence compliance, or place the patient at increased risk, or otherwise make them unsuitable for participation

Orthopedic surgeons perform an initial screening of all patients referred to the department for surgery. If the patients fulfill the eligibility criteria they are referred to the principal investigator, who performs the final eligibility assessment, give the patient detailed information about the study and ask for consent to participate in the study. If the patient consents to participate a baseline assessment visit is performed between 1 and 14 days before surgery, on the understanding that the patient is not finally included and allocated until the surgeon verifies a total and repairable supraspinatus tear. After surgery the orthopedic surgeon provides surgery information (verifying reparable tendon (s)), and included patients are electronically randomized (described in “Allocation, randomization and sequence generation”).

The rehabilitation sessions are conducted at the local physiotherapy departments by 10 orthopedic trained physiotherapists with postgraduate musculoskeletal experience between 8 and 25 years, who have been thoroughly trained in the rehabilitation protocols, including the different phases, restrictions and progression.

### Interventions

Patients in both treatment groups have the affected shoulder immobilized in a fixed sling for 2 weeks followed by 3 weeks in a non-fixed sling. The PR group starts loading (assisted active range of motion (AAROM) and active range of motion (AROM)) from week 2, while this is introduced in the UC group from week 6. The PR group is attending individual physiotherapist-supervised exercise therapy three times weekly, supplemented with daily home exercises (week 2, 3, 4 and 5), and the UC group is attending individual physiotherapist-supervised exercise therapy once a week supplemented with daily home exercises (week 2, 3, 4 and 5). From weeks 6 to 12 all patients receive physiotherapist-supervised exercise therapy twice a week (individually or in small groups) next to the self-administered exercise once a week (Table 1). Descriptions of on-site and home interventions are found in the additional files (Additional files 1 and 2).

**Table 1:** Overview of the postoperative intervention in main trial

Week	Progressive rehabilitation (PR)	Week	Usual care (UC)
1- 5	Shoulder immobilized in standard sling.	1- 5	Shoulder immobilized in standard sling.
2- 5	Physiotherapist guided PROM exercises PROM Restrictions: ABD + FLEX: None IR < 90 degrees in neutral ER < 45 degrees in neutral	2- 5	Physiotherapist guided PROM exercises PROM Restrictions: ABD + FLEX: None IR < 90 degrees in neutral ER < 45 degrees in neutral
2	Close-chain AAROM and AROM exercises AAROM and AROM Restrictions: ABD + FLEX < 90 degrees IR < 90 degrees in neutral ER = 0 degrees in neutral		
3- 5	Close-chain AAROM and AROM exercises AAROM and AROM Restrictions: ABD + FLEX < 90 degrees IR < 90 degrees in neutral ER < 45 degrees in neutral		
6-12	Therapist-supervised AROM (FLEX, ABD, EXT, ER and IR) with gradually (individually) increased loading and progression from close-chain to open-chain exercises.	6-12	Therapist-supervised AROM (FLEX, ABD, EXT, ER and IR) with gradually (individually) increased loading and progression from close-chain to open-chain exercises.
12-20	Continuation of rehabilitation in the community	12-20	Continuation of rehabilitation in the community

*PR* progressive rehabilitation, *UC* usual care, *ROM* range of motion, *PROM* passive range of motion, *ABD* abduction, *FLEX* flexion, *IR* Internal rotation, *ER* external rotation, *AAROM* assisted active range of motion, *AROM* active range of motion, *EXT* extension

The exercise therapy program is tailored to each patient’s capabilities at any given session. The therapists adjust exercise intensity as determined by the patient’s ability to complete 3 sets of 10 repetitions for a given exercise without exacerbating pain. The overriding rule for all exercises is that pain above 5 on a numeric pain rating scale (NPRS) from 0 to 10 should not be provoked during exercises. The exercise therapy continuously determines and applies the load for 20 repetitions maximum (RM), with progression from ½ kg to 3–4 kg during the 12 weeks. Each exercise is guided with focus on correct performance and movement quality (direction, speed, posture and coordination) with sufficient rest between sets to allow for recovery.

It is recommended to increase the load by 2–10% when the patient can perform the current workload properly and with 1–2 repetitions more than the required number of 10 repetitions (Additional file 2). For both groups, scapular exercises are also performed with 20 RM, while stretching and mobility exercises are performed five times, for 20 s each. Both groups are instructed to complete a diary during the supervised sessions, including self-reported pain and use of pain medication as registered before and after each session.

### Treatment adherence

Intervention adherence and attendance (on site and home-based) within the 12 weeks is recorded in exercise logbooks for both groups. In the exercise logbook, the patients are asked to report completed home-based exercise sessions and reasons for non-completed sessions (pain or other reasons). Supervision of the subsequent home exercises at the commencement of every session is performed to facilitate program adherence. Reinforcement techniques are used with the physiotherapist giving positive feedback and commending patients for their efforts.

As part of the post-operative standard medical treatment package all patients are offered paracetamol and non-steroidal anti-inflammatory drugs (NSAIDs) as basic pain treatment supplemented with morphine if necessary, and medication is prescribed before inclusion and randomization. Concomitant private physiotherapy sessions, acupuncture or other healthcare initiatives are prohibited during the trial. The available exercise equipment during the supervised rehabilitation includes Steens Physical Double Handle Speed Pulley (weight interval 0.5–2 kg) (Proterapi), Thera-Band System of progressive exercise, carpet tile, jump rope and broomstick. Also, sloping board (for supine elevation exercise in the scapular plane), ProFitter and various vinyl-coated hand weights produced by Trendy Sport will be available.

### Outcome measures/variables

**Fig. 2:**
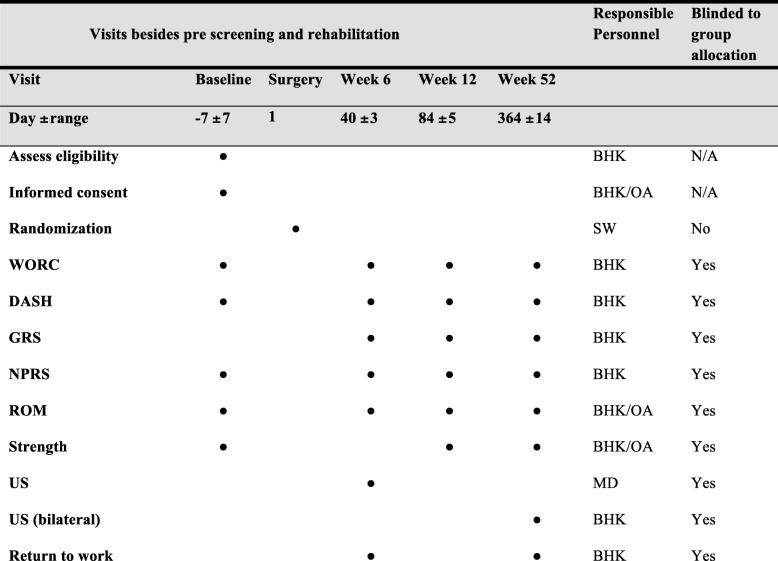
Time schedule of enrollment, assessments and responsible trial personnel (Standard Protocol Items: Recommendation for Interventional Trials (SPIRIT)). BHK, primary investigator; SW, co-author; N/A, not applicable; OA: outcome assessors; MD, medical doctor/ radiologist; WORC, Western Ontario Rotator Cuff Index; DASH, Disabilities Arm, Shoulder and Hand questionnaire; MRI, magnetic resonance imaging; GRS, Global Rating Scale; NPRS, numeric pain rating scale; ROM, range of motion; US, ultrasound

The primary (Western Ontario Rotator Cuff index, (WORC)) and secondary outcomes (Disability Arm Shoulder Hand (DASH), pain, ROM) are used at baseline (pre-surgery), 6 weeks, 12 weeks and 12 months after baseline (post-surgery) in addition to measurements of strength at baseline, 12 weeks and 12 months after baseline, and US measurements only 12 months after baseline (Fig. 2).

The primary outcome is the 12-week change in the WORC [47]. The WORC is a self-administered questionnaire developed (by interviewing patients) to measure health related quality of life (HRQL) in patients with rotator cuff disorders and it consists of 21 items in 5 domains: physical symptoms (6 items), sports and recreation (4 items), work (4 items), lifestyle (4 items) and emotions (3 items). Each question is scored on a 100 mm visual analogue scale (VAS) and summed to a total score for each domain, with a higher score indicating reduced quality of life. By inverting the raw score and then converting it to a percentage score ((2100–1875)/2100 × 100 = 10.7%)) each domain ranges from 0 (worst possible) to 100 (best possible) [47, 48]. The WORC has excellent reliability with intra class correlation (ICC) between 0.84 and 0.99 [49, 50] and a minimal clinical important difference (MCID) of approximately 12% [47, 49, 51]. A recent translated and cross-culturally adapted Danish version was found valid in patients with rotator cuff tear (high correlation with the DASH (0.71), and test re-test reliable (ICC of 0.80 95% CI 0.69–0.87)) [52].

Secondary patient-reported outcomes (PRO) include assessment of pain, functional activity level and HRQOL, using the DASH [53]. The DASH questionnaire is a self-administered questionnaire and is region-specific to upper extremity disorders and consists of 30 items divided into 6 items on symptoms and 24 on function. The questionnaire score ranges from 0 to 100 where 0 equals no disability and 100 equals the most severe disability [53]. The DASH is generated by asking questions of patients with upper extremity problems/disability and is valid, reliable and responsivene for this patient group [53, 54]. The Danish validated version was to found to be a reliable [55] and responsive outcome measure in a variety of Danish-speaking patients with orthopedic upper extremity problems [56].

The Patient Global Rating Scale (GRS) is used to obtain a global/general impression of recovery from baseline to 6, 12 and 52 weeks postoperatively with the question: “Compared to when this treatment first started (before surgery), how would you describe your shoulder this last week?” This is assessed on a 15-point scale where − 7 represents vastly worse, 0 represents unchanged, and + 7 represents much better [57–59].

Patients are also asked about perceived pain using a NPRS [60, 61] with three questions: “On a scale from 0-10, where 0 equals no pain and 10 the worst imaginable pain, how much pain do you feel in your shoulder during resting? / How much pain do you feel in your shoulder during activity? / What is the maximal shoulder pain you have experienced within the past 24 hours?”

Further, patients are asked to report use of analgesics (type (paracetamol or opioids)/amount). Secondary clinical outcomes consist of active and passive shoulder elevation ROM in the scapular plane (standing); external and internal shoulder rotation (in 90° abduction when supine) [62, 63], as measured by digital inclinometer (Baseline Evaluation Instruments, model 12–1057 from Procare). Maximum voluntary isometric contraction (MVC) of shoulder external and internal rotation (sitting) and elevation in the scapular plane [62, 64, 65] is measured by dynamometer (IsoForceControl, model EVO2, 10-400 N, Medical Device Solutions AG). Furthermore, intraoperative information (including details of tendon repair) and patient-reported number of sick-leave days from work/time until return to work and leisure activities are registered.

Tendon healing characteristics of the repaired supraspinatus tendon (SST) and the subacromial space (SAS) are investigated at 12 months follow-up using US greyscale. The SAS and the SST thickness are also potentially important factors in understanding the pathogenesis of rotator cuff pathology [66, 67], tendinopathy [68] and ruptures [69] a year after repair [70]. Furthermore, power Doppler (PD) US is used for measuring vascularization as a sign of pathology and healing [70, 71]. The US examination is performed by the principal investigator (BHK) using a Hitachi Ascendus scanner and Hitachi Medical Systems Linear standard probe type L75 18–5 MHz 50-mm transducer (Hitachi Aloka Medical) and the musculoskeletal program. The depth preset is 2.50 cm and focus is adjusted, so it is placed right under the tendon.

Thus, the US investigation at 12 months involves quantitative measures of the repaired and contralateral SST thickness and neovascularity (NV), in addition to the SAS measured as the acromiohumeral distance (AHD). Ultrasound images are captured with the patients seated in an upright position, feet flat on the floor, neutral trunk posture, and head facing forward. For the SST thickness measure the patient has the hand on the affected side on the ipsilateral posterior hip with the humerus in extension and the tendon is measured in a longitudinal [72] and a transverse view [67]. AHD is measured in the neutral position (arm resting at the side) and respectively with 45° and 60° of active shoulder elevation in the scapular plane [72, 73]. SST neovascularity assessed by power Doppler is measured with the shoulder internally rotated with the dorsal side of the hand placed on the sacrum, and the elbow flexed and directed laterally [74].

Registered demographic data include age, gender, tendon(s) involved, supplementary surgery performed, hand dominance, history of trauma, occupation/employment and preoperative sports/recreational activity level. Demographic data and baseline measures will be used in the analysis of predictors of health outcomes.The investigator or site personnel monitor each patient for adverse events (AEs) throughout the study. Tendon re-tear is assessed by US at 6 weeks postoperatively and any re-tear is recorded. The investigator assesses and records any AE (in response to a query, observed by site personnel or reported spontaneously by the patient) in detail, including the date of onset, description, severity, duration and outcome, relationship of the AE to study intervention and any action(s) taken. All AEs will be followed closely to make sure that the patient is safely handled including follow up on clinical issues, until a stable situation is reached.

### Data collection

The primary investigator performs all enrollment after screening by the orthopedic surgeons. The primary investigator and two outcome assessors perform all baseline and follow-up assessments. Before starting the data collection, the assessors and the primary investigator decide on a consensus standard for collection and recording of all outcome variables. We use the Procordo Research Platform, which is an electronic online data trial management system (DTMS) (www.procordo.com) to collect and store the data. In the system the patients answer the questionnaires in a web-based survey form, and the outcome assessor manually registers data from the objective assessments. The DTMS includes automatic range checks of the entered data values, and the user will be alerted if the entered data fall outside a pre-specified range.

### Sample size and power considerations

The sample size is calculated to test the superiority of the PR protocol over the UC protocol in the assessment of change in the WORC physical symptoms subscale at week 12 (primary outcome) [75]. With 41 patients per group, the study will have 80% power assuming the expected group difference in the mean changes from baseline is 12 points, corresponding to the suggested minimum clinical relevant difference [47], the common standard deviation is 20 (0–100 scale) [75] and a significance level of 5%. With an expected dropout around 20% during the study we will randomize and allocate 100 patients (50 to each group); analyzing the intention to treat (ITT) population, which will give power of 85% to detect the aforementioned difference. A provisional deadline for patient recruitment is set to June 2018, but in case the target number of 100 patients has not been met the recruitment period may be extended to reach the number required (2 times 41 patients) to obtain power of at least 0.8 (80%).

### Allocation, randomization and sequence generation

After baseline tests and surgery the patients will be randomized equally (1:1) to receive either the PR protocol or the UC protocol. The computer-generated randomization to one of two treatment arms is performed based upon permuted random blocks of variable size (4–8 in each block) using the Procordo Research Platform. To counter potential imbalance in the randomization, stratification by trial site and age (+/− 57 years) will be employed together with blocking. The randomization is performed by a person (SW) with no clinical involvement in the trial, and the person notifies the patient and clinical study staff of the treatment allocation. The allocation will be concealed in a password-protected Research Platform only accessible by the senior researcher and the independent data manager (Procordo).

### Blinding

Orthopedic surgeons will perform initial screening. As this is an “open-label” trial the health professionals delivering the interventions and the patients will not be blinded to treatment allocation. The principal investigator and outcome assessors will be blinded to treatment allocation during all pre-examinations and post-examinations, and patients are requested not to disclose their allocation when outcomes are assessed at weeks 6 and 12 and 12 months postoperatively. To test the efficacy of blinding, the outcome assessors are asked what treatment strategy they think a patient has received after assessments at week 12 postoperatively. The principal investigator and outcome assessors can be un-blinded if deemed necessary, e.g. in the case of (serious) adverse events that require these otherwise blinded persons to be involved in the solution of the event.

### Statistical analysis plan

The primary efficacy analysis performed is assessment of the between-group difference in change in the WORC score after 12 weeks in the ITT population. The ITT population is defined as all randomized patients irrespective of compliance or withdrawals. Missing data will be replaced using a multiple imputation technique with age, gender, center, group allocation (masked) and baseline values as predictors. For sensitivity purposes missing data will be imputed using baseline observation carried forward (BOCF). The modified ITT population is defined as the ITT population that has a valid baseline observation of the variable of interest. The as-observed (AO) population is defined as patients who have the outcome of interest assessed at a given time point of interest (i.e. no imputation of missing data will be done).

The per-protocol (PP) population is defined as patients in the AO population that adhere to this protocol, defined by the criteria of the two groups: the PR group (Table 1), included in the AO population and have attended at least 75% of the scheduled rehabilitation appointments, whether at clinical visits or home-based as tailored to the individual (documented by the exercise logbook), and do not stop the intervention during the 12-week main trial period and do not engage in concomitant exercise therapy (e.g. private physiotherapy sessions, acupuncture); and the UC group (Table 1), included in the AO population, and do not stop the intervention during the 12-week main trial period and do not engage in concomitant exercise therapy. Each patient registers the home shoulder exercises in an exercise logbook, and successful completion of the intervention is defined as performance of 75% of the planned exercises.

The primary analysis at week 12 will be by analysis of covariance (ANCOVA) on change from baseline in the WORC domains as dependent variables, with group allocation (two levels) and time (week 6 and 12), gender and site as fixed factors, whereas age and baseline values will be analyzed as covariates. Secondary outcome measures will be analyzed by multiple logistic regression for binary outcome measures (re-tear, tendon healing, recurrence of symptoms (e.g. pain and function), complications) and by ANCOVA for the continuous outcome measures (pain, patient-reported outcomes, surgery information, strength, ROM) as dependent variables (individually), with group allocation and time as fixed factors, and site and baseline values as covariates. Predictors of health outcomes will be analyzed by ANCOVA or multiple regression as independent variables, with change in the WORC as the dependant variable. All statistical tests will be two-sided and statistical significance will be denoted by a computed *p* value equal to or less than 0.05. All data analysis will be carried out according to a pre-established analysis plan. The statistical analysis plan (SAP) will be carried through blinded according to group allocation, and results will be interpreted in an author consensus statement prior to disclosing/revealing group allocation on the basis of a blinded review of the data from the primary endpoint (changes from treatment A compared to changes from treatment B), assuming that treatment A is the active treatment (PR), and the other assuming that treatment B is the active treatment (UC). Analysis of the primary outcome will be calculated blinded, and two interpretations will be performed based on whether PR or UC is superior. Not until signed consent from all of the authors of this trial (identical to the authors of this SAP) has been obtained, agreeing on one interpretation of the results only, will the randomization code be broken. This is to reduce bias in the interpretation of the current findings. On agreement, all members of the author group will approve and sign the interpretations before any publication procedures are initiated [76].

The protocol conforms to the recommendations of the Enhancing the Quality and Transparency Of health Research (EQUATOR) network [77] using the Standard Protocol Items: Recommendations for Interventional Trials (SPIRIT) checklist and the Consolidating Standards of Reporting Trials (CONSORT) statement [78] (Additional file 3).

### Interim analysis and early stopping rules

An early stopping rule will be applied when there is complete rotator cuff re-tears corresponding to at least 25% of the calculated number in the PR group as verified by ultrasonography 6 weeks postoperatively, provided that the re-tear rate is more than twice as high compared to that in the control UC group [79]. The trial steering committee (the authors of this manuscript) decide if the early stopping rule should be effectuated.

### Safety monitoring

No data monitoring committee has been established since adverse events are expected to be minimal and the intervention is not considered to be high risk. The physiotherapists at each center will report any adverse event to the primary investigator, who reports these to the ethics committee. The person responsible for randomization will monitor if the number of adverse events has reached the threshold for the early stopping rule. Further, if a patient experiences extreme worsening of symptoms during exercise therapy and these symptoms have not subsided before the next exercise session, the physiotherapist will report it to the primary investigator, who may refer the patient to an orthopedic specialist for an evaluation. The number and seriousness of AEs will be reported.

### Ethics

Prior to screening, all potential trial patients are informed orally and in writing about the purpose of this trial, its process and potential risks and the costs and benefits of participation. All patients are informed of their rights to withdraw from the study at any time without this impacting on any future investigations and/or treatments at any site or by any member inside or outside the study staff. Informed consent will be obtained from all patients. All data will be handled in confidence according to the Danish Data Protection Act. The study was approved by Health Research Study Board for the Capital Region Denmark (H-16033995) on the 18 October 2016 and by the Danish Data Protection Agency (2012–58-0004) on the 15 February 2017 and the study was registered at www.clinicaltrials.gov (NCT02969135) on the 15 November 2016. The study will be conducted in accordance with Danish law, the local research ethics committee requirements and the principles of the Declaration of Helsinki [80]. Positive as well as negative and inconclusive results will be published in scientific peer-reviewed journals, with authorship following the International Committee of Medical Journal Editors (ICMJE) guidelines for publication. The results will be presented orally nationally and internationally. Upon full publication of the dataset by the investigators, we intend to share the data for future research purposes.

## Discussion

The effect of rehabilitation after rotator cuff surgery has been discussed during the past 10 years and 13 systematic reviews including meta-analyses or systematic review of overlapping meta-analyses have been published since 2014 including the same few (8–12) primary studies [21–26, 81–87]. These have focused on early ROM exercises and immobilization periods. Nevertheless, there are only a few clinical studies on postoperative treatment of individuals with rotator cuff repair and the existing studies are of moderate to low quality. This is primarily due to small sample sizes, inadequate blinding of patients and/or investigators and incomplete intervention descriptions [15–20], making it difficult to transform the results into clinical practice. Furthermore, previous studies use a wide range of clinical and patient-reported outcomes, generally with poor responsiveness and per-protocol analyses. However, collectively there is evidence that early intervention is advantageous in the rehabilitation because early ROM exercises accelerate healing, reduce stiffness, do not increase risk of re-tear, and because immobilization does not increase tendon healing or clinical outcome [21–24].

The CUT-N-MOVE trial will add new knowledge to the field with regards to the effect of an early and targeted progressive treatment program for patients who had surgery due to a rotator cuff tear. To our knowledge this study is the first evaluating the combined effect of early (time of initiation) and progressive (intensity, also including active muscle contraction) postoperative exercises on parameters such as physical function, pain, quality of life and return to work.

Inclusion criteria in this study are broad with respect to age and concurrent diseases in an attempt to reflect clinical practice. However, inclusion criteria are narrow regarding the specific condition, and thus we require the tears to be based on relevant extrinsic trauma and at the least a complete supraspinatus tear must be verified during surgery/arthroscopy.

The present exercise program focuses in several ways (passive, assisted active and active ROM exercises and closed and open chain exercises) on each individual patient’s capability on the day. Further, this program differs, to our knowledge, from previously outlined exercise programs in having progressive but gently assisted active exercise starting as soon as the second week postoperatively. Importantly, in addition to functional outcomes (considered the most relevant for the patients) we also follow the pathological and mechanical tendon-healing process compared with the contralateral side.

The aim of the CUT-N-MOVE trial is to investigate whether there is a significant advantage in using progressive early passive and active exercise therapy compared with a traditional, limited early passive exercise therapy in patients recovering from surgical treatment of rotator cuff tears. If this is the case, the consequence is that clinicians will have a detailed description of a step-wise progressive rehabilitation program available when treating patients who have had surgery due to a rotator cuff tear.

### Trial status

At the time of submission of this study protocol the trial is ongoing and still recruiting patients. The recruitment of patients began in February 2017 and will continue until the complete sample size is achieved, which is expected in June 2018.

## Additional files

Additional file 1: Exercise therapy program for the CUT-N-MOVE trial. (PDF 53848 kb)

Additional file 2: Exercise progression for the CUT-N-MOVE trial. (PDF 234 kb)

Additional file 3: The SPIRIT checklist for the CUT-N-MOVE trial. (PDF 99 kb)

## Abbreviations

AAROM: Assisted active range of motion
ABD: Abduction
AE: Adverse event
AHD: Acromiohumeral distance
ANCOVA: Analysis of covariance
AO: As observed
AROM: Active range of motion
BOCF: Baseline observation carried forward
CONSORT: Consolidating Standards Of Reporting Trials
DASH: Disabilities Arm, Shoulder and Hand questionnaire
EQUATOR: Enhancing the quality and transparency of health research
ER: External rotation
EXT: Extension
FLEX: Flexion
GRS: Global Rating Scale
HRQOL: Health related quality of life
ICC: Intra class correlation
IR: Internal rotation
ITT: Intention to treat
MCID: Minimal clinical important difference
MVC: Maximal voluntary contraction
NPRS: Numeric Pain Rating Scale
NV: Neovascularity
PD: Power Doppler
PP: Per protocol
PR: Progressive rehabilitation
PRO: Patient-reported outcomes
PROM: Passive range of motion
RC: Rotator cuff
ROM: Range of motion
SAP: Statistical analysis plan
SAS: Subacromial space
SPIRIT: Standard Protocol Items: Recommendation For Interventional Trials
SST: Supraspinatus tendon
UC: Usual care
US: Ultrasound
VAS: Visual analogue scale
WORC: Western Ontario Rotator Cuff Index
