# Supplementary material for: Progressive early passive and active exercise therapy after surgical rotator cuff repair – study protocol for a randomized controlled trial (the CUT-N-MOVE trial)
Source: Trials. 2018 Sep 3;19:470. doi: 10.1186/s13063-018-2839-5 (PMC6122575; doi:10.1186/s13063-018-2839-5)
Supplement: Supplementary file 1 — Exercise therapy program for the CUT-N-MOVE trial. (PDF 53848 kb) [file 13063_2018_2839_MOESM1_ESM.pdf]

Bispebjerg and Frederiksberg Hospital

Department of Physical and Occupational Therapy

Exercise therapy program for the CUT-N-MOVE trial

Developed by Birgitte Hougs Kjær PT, MSc, PhD Fellow, Bente Hannibal PT & Rikke Høffner PT,
Orthopaedic Department and Department of Physical and Occupational Therapy,
Bispebjerg Frederiksberg Hospital, Copenhagen.

The exercise therapy program and concurrent use of the dotsheets with exercise progression (additional file 2) is intended as a guide for the PT responsible for the supervised exercise therapy in the CUT-N-MOVE trial. The program contains a varying number of exercises targeting the glenohumeral and scapular stabilizing muscles with gradual loading the rotatorcuff tendons.

The exercise therapy program is tailored to each patient´s capabilities at any given session. The therapists adjust exercise intensity as determined by the patient´s ability to complete 3 sets of 10 repetitions for a given exercise without exacerbating pain. The overriding rule for all exercises is that pain above 5 on a NPRS (Numeric Pain Rating Scale) from 0 to 10 should not be provoked during exercises.

Exercise equipment available during the supervised exercise therapy: Follo Diem Double Handle Pulley (weight interval 0.5- 2 kilo), Thera-Band System of progressive exercise, carpet tile, jump rope, foam block, broomstick, sloping board, ProFitter and various vinyl coated hand weights.

Development of the exercise therapy program

The exercise therapy program was developed based on existing literature, in which shoulder muscle activity and function in shoulder rehabilitation exercises was described, including electromyographic analysis of specific exercises for rotatorcuff- and scapular muscles in early phases of shoulder rehabilitation and literature on rehabilitation following rotator cuff repair (1-20). Moreover, the basis of this exercise program is formed from neuromuscular theories, exercise experience from clinical practice with input from clinical physiotherapists with theoretical and practical knowledge of neuromuscular training aspects.

References

1. Yi A, Villacis D, Yalamanchili R, Hatch GF, 3rd. A Comparison of Rehabilitation Methods After Arthroscopic Rotator Cuff Repair: A Systematic Review. Sports health. 2015;7(4):326-34.
2. Shen C, Tang ZH, Hu JZ, Zou GY, Xiao RC, Yan DX. Does immobilization after arthroscopic rotator cuff repair increase tendon healing? A systematic review and meta-analysis. Archives of orthopaedic and trauma surgery. 2014;134(9):1279-85.
3. Parsons BO, Gruson KI, Chen DD, Harrison AK, Gladstone J, Flatow EL. Does slower rehabilitation after arthroscopic rotator cuff repair lead to long-term stiffness? Journal of shoulder and elbow surgery / American Shoulder and Elbow Surgeons [et al]. 2010;19(7):1034-9.
4. McClure PW, Michener LA. Staged Approach for Rehabilitation Classification: Shoulder Disorders (STAR-Shoulder). Physical therapy. 2015;95(5):791-800.
5. Maenhout A, Van Praet K, Pizzi L, Van Herzeele M, Cools A. Electromyographic analysis of knee push up plus variations: what is the influence of the kinetic chain on scapular muscle activity? British journal of sports medicine. 2010;44(14):1010-5.
6. Ludewig PM, Hoff MS, Osowski EE, Meschke SA, Rundquist PJ. Relative balance of serratus anterior and upper trapezius muscle activity during push-up exercises. The American journal of sports medicine. 2004;32(2):484-93.
7. Long JL, Ruberte Thiele RA, Skendzel JG, Jeon J, Hughes RE, Miller BS, et al. Activation of the shoulder musculature during pendulum exercises and light activities. The Journal of orthopaedic and sports physical therapy. 2010;40(4):230-7.
8. Littlewood C, Bateman M, Clark D, Selfe J, Watkinson D, Walton M, et al. Rehabilitation following rotator cuff repair: a systematic review. Shoulder & Elbow. 2015;7(2):115-24.
9. Kuhn JE. Exercise in the treatment of rotator cuff impingement: a systematic review and a synthesized evidence-based rehabilitation protocol. Journal of shoulder and elbow surgery / American Shoulder and Elbow Surgeons [et al]. 2009;18(1):138-60.
10. Kluczynski MA, Nayyar S, Marzo JM, Bisson LJ. Early Versus Delayed Passive Range of Motion After Rotator Cuff Repair: A Systematic Review and Meta-analysis. The American journal of sports medicine. 2015;43(8):2057-63.
11. Kluczynski MA, Isenburg MM, Marzo JM, Bisson LJ. Does Early Versus Delayed Active Range of Motion Affect Rotator Cuff Healing After Surgical Repair? A Systematic Review and Meta-analysis. The American journal of sports medicine. 2016;44(3):785-91.
12. Kibler WB, Sciascia AD, Uhl TL, Tambay N, Cunningham T. Electromyographic analysis of specific exercises for scapular control in early phases of shoulder rehabilitation. The American journal of sports medicine. 2008;36(9):1789-98.
13. Keener JD, Galatz LM, Stobbs-Cucchi G, Patton R, Yamaguchi K. Rehabilitation following arthroscopic rotator cuff repair: a prospective randomized trial of immobilization compared with early motion. The Journal of bone and joint surgery American volume. 2014;96(1):11-9.
14. Hardwick DH, Beebe JA, McDonnell MK, Lang CE. A comparison of serratus anterior muscle activation during a wall slide exercise and other traditional exercises. The Journal of orthopaedic and sports physical therapy. 2006;36(12):903-10.
15. Escamilla RF, Yamashiro K, Paulos L, Andrews JR. Shoulder muscle activity and function in common shoulder rehabilitation exercises. Sports medicine. 2009;39(8):663-85.
16. Ellenbecker TS, Cools A. Rehabilitation of shoulder impingement syndrome and rotator cuff injuries: an evidence-based review. British journal of sports medicine. 2010;44(5):319-27.
17. Cricchio M, Frazer C. Scapulothoracic and scapulohumeral exercises: a narrative review of electromyographic studies. Journal of hand therapy : official journal of the American Society of Hand Therapists. 2011;24(4):322-33; quiz 34.
18. Cools AM, Struyf F, De Mey K, Maenhout A, Castelein B, Cagnie B. Rehabilitation of scapular dyskinesis: from the office worker to the elite overhead athlete. British journal of sports medicine. 2014;48(8):692-7.
19. Cools AM, Dewitte V, Lanszweert F, Notebaert D, Roets A, Soetens B, et al. Rehabilitation of scapular muscle balance: which exercises to prescribe? The American journal of sports medicine. 2007;35(10):1744-51.
20. Boettcher CE, Ginn KA, Cathers I. Which is the optimal exercise to strengthen supraspinatus? Medicine and science in sports and exercise. 2009;41(11):1979-83.

Exercise #	Exercise Name	Start position	Action	Description
Part 1 Tendon protection				
A	Active elbow extension (supine/ standing)	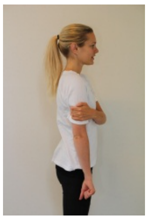		Extraordinarily, the forearm must be removed from the sling during this exercise. While standing, slowly flex and extend the elbow with the foream sliding along the trunk. In supine keep the elbow fully extended for a little while.
B	Vein pump (spread/collect fingers)	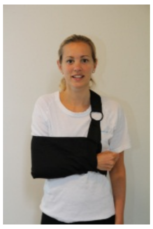	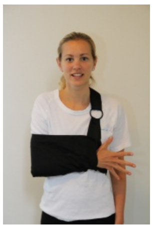	Spread and collect the fingers to avoid or reduce swelling.
C	Rotate forearm (supination/ pronation)	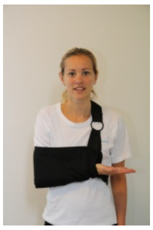	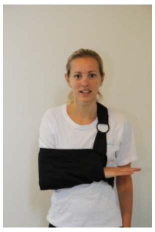	Rotate the forearm alternately from supination to pronation.
D	Lift sternum (retraction of shoulders)	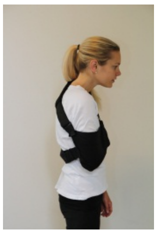	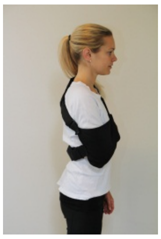	Stand up straight and lift the sternum. Retract the shoulders. Can be done in supine.
E	Head lateral flexion	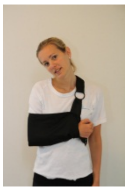	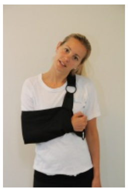	Lateral flexion of the head, moving the ear towards the shoulder. Performed bilaterally. Feel a stretch on both sides of the neck.
F	Cervical flexion	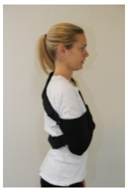		Make a long neck by retracting the chin. Stand up straight and lift the sternum. Can be done in supine.

Part 2 Passive range of motion (by PT)				
1	Flexion	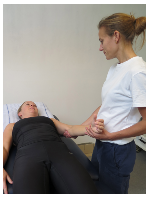	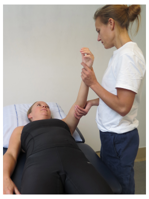	First perform the movement on the healthy side.<br>Ask the patient to relax the arm to allow passive flexion.<br>The movement is performed until the patient says stop or tissue resistance is felt by the PT.
2	Abduction	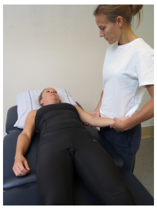	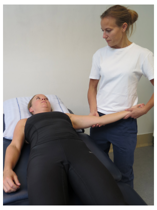	First perform the movement on the healthy side.<br>Ask the patient to relax the arm to allow passive abduction.<br>The movement is performed until the patient says stop or tissue resistance is felt by the PT.
3	Supine external rotation w.<br>20-60 deg. abd.	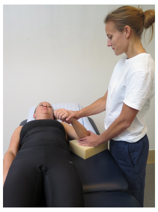	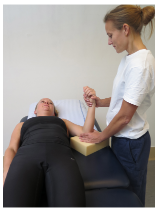	First perform the movement on the healthy side.<br>Ask the patient to relax the arm to allow passive external rotation.<br>The movement is performed until the patient says stop or tissue resistance is felt by the PT.<br>Note: not subscapularis tear.
4	When needed manual guiding of scapula	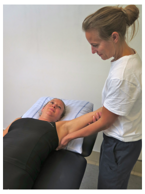		During abduction upward rotation of the scapula can be guided manually by grasping the inferior angle. The movement is performed until the patient says stop or tissue resistance is felt by the PT.
5	When needed manual therapy	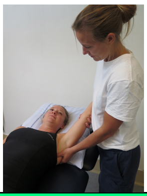		Manual stretching of the subscapularis and the latissimus can be performed during full flexion.
Part 2 Assisted active range of motion				
6	Reach the foot (flexion)	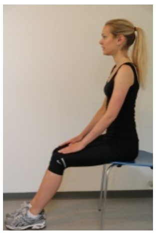	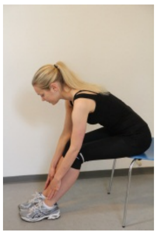	Sit up straight and place a hand on each thigh. Lean forward while sliding the hands down the legs.
7	Wash the table (flexion on table)	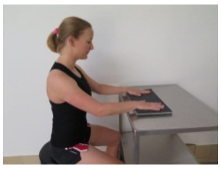	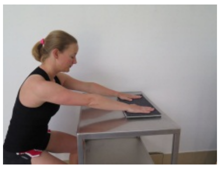	Sit facing a table. Place the hands on a piece of cloth. Slide the cloth forth and back, from side to side and in circles.<br>Note: A big ball on the flor can also be used.

8	Supine bench press w/ broomstick	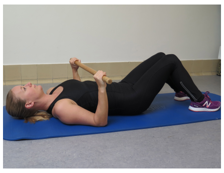	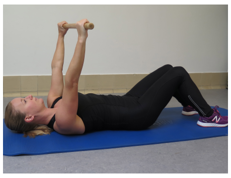	Extend the arms up in the air.<br>If no restrictions the arms can also be extended over the head.
9	Flexion/ scaption w/ jump rope	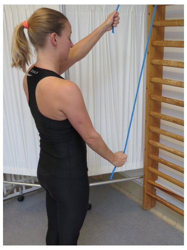	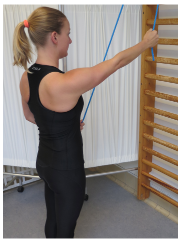	Face the rib. Stand up straight and lift the sternum. The rope is placed around the top rib. Pull down with the healthy arm thus flexing the operated arm.<br>Can also be performed with abduction.<br>Note: Keep the shoulders down.
10	Abduction w/ broomstick	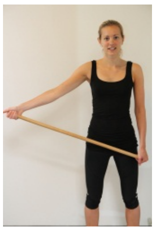		Stand up straight and lift the sternum. Hold the broomstick, pointing the thumb of the operated arm upwards.<br>Let the healthy hand push the broomstick to the side.
11	Supine external rotation w/ broomstick	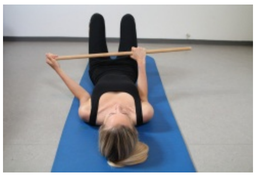		Supine with 90 deg. flexed elbows.<br>Hold the broomstick in the hand of the operated arm and push the forearm to the side with the healthy hand.<br>Maintain neutral wrists.<br><br>Note: Not subscapularis tear.
Part 2 Active exercise closed and open chain				
12	Downward pressure in abduction	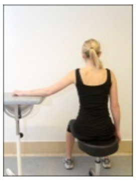	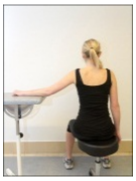	Sit up straight and rest the forearm on a table with the shoulder in abduction. Tie your hand and pres it down against the table.<br>At the same time depress the scapula.<br><br>Hold the position for 5 sec.
13	Low row w/ Thera-Band (extension)	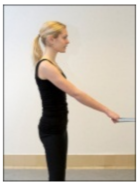	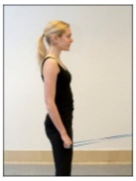	Stand up straight and lift the sternum. The shoulder is flexed 45 deg. with extended elbow.<br>Pull the theraband towards extension until the hand reaches the hip. Slowly return the arm to the starting position.
14	Incline plinth sliding (flexion)	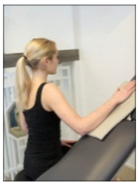	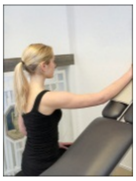	Sit up straight next to the incline plinth with the hand resting on a cloth.<br>Slide the hand upwards and return the arm to the starting position.

15	Sitting bilateral downward press	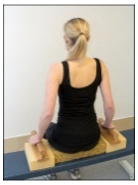	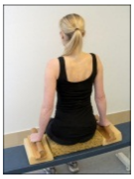	Sit on a bench with the feet on the ground. The hands are placed on the handles. Position the scapulae. Pres down on handles, extending elbows. Keep the back straight and avoid lifting the shoulders.
16	High row with pulley	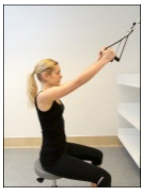	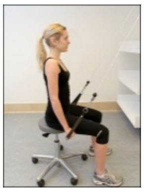	Sit facing pulley. Start with arms extended in front of the body. Pull down toward extension.<br>Keep the back straight and avoid lifting the shoulders.<br>Note: Can be performed with a long Theraband.
17	Fitter	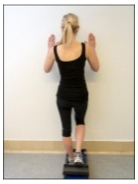	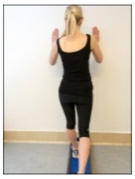	Stand on Fitter facing the wall.<br>Hands are placed on the wall at shoulder level. Protract the shoulders pushing the thorax away from the wall. Move one leg backwards.
18	Prone low row	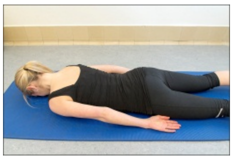	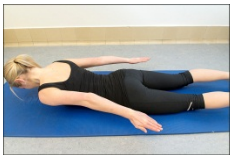	Prone with arms extended in internal rotation. Lift thorax from the matress and extend and external rotate the shoulders squeezing scapulae together.
19	Supine scaption on sloping board	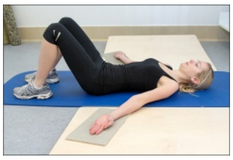	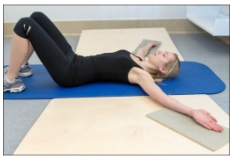	Supine with each arm extended on a sloaping board. Hands lying in supination on a carpet tile. Alternately, slide into shoulder abduction and back.
20	Supine internal rotation w/ pulley	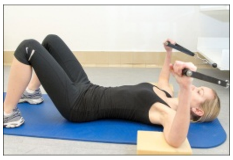	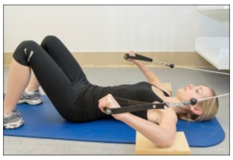	Supine with feet away from pulley. Arms are placed on a foam block in 90 deg. shoulder abduction. Elbows flexed 90 deg.<br>Pull forward into shoulder internal rotation.<br><br>Note: Can be performed with a long Theraband.
21	Supine external rotation w/ pulley	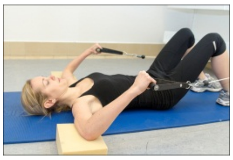	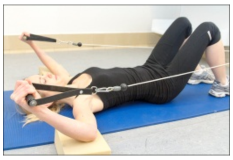	Supine with feet facing pulley. Arms are placed on a foam block in 90 deg. shoulder abduction. Elbows flexed 90 deg.<br>Pull backwards into shoulder external rotation.<br>Note: Can be performed with a long Theraband.

22	Standing internal rotation w/ pulley	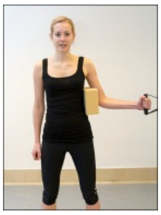	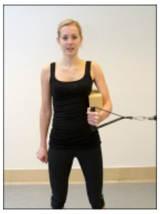	Standing with operated shoulder next to pulley. Stand up straight and lift the sternum. Elbow flexed 90 deg. and shoulder in external rotation. Foam block between elbow and trunk.<br>Pull into internal rotation.<br><br>Note: Can be performed with a long Theraband.
23	Lawn mover	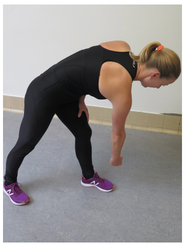	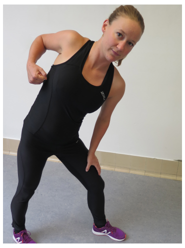	Standing one foot in front, leaning down and across body towards opposite foot. Step back and move arm into abduction / external rotation, action as if starting a lawn mower.<br><br>Note: Can be performed with Theraband or light hand weight.
24	Standing external rotation w/ pulley	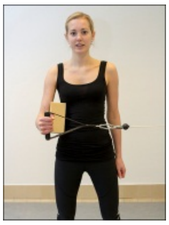	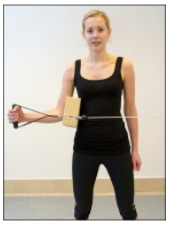	Standing with healthy shoulder next to pulley. Stand up straight and lift the sternum. Elbow flexed 90 deg. and shoulder in internal rotation. Foam block between elbow and trunk.<br>Pull into external rotation.<br><br>Note: Can be performed with a long Theraband.
25	Wall slide	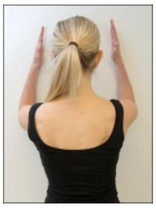	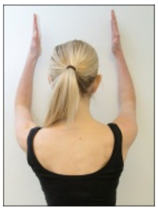	Stand facing wall with feet 15 cm. from the wall. Shoulders and elbows in 90 deg. flexion, ulnar side of forearms placed on the wall with neutral shoulder rotation. Alternately, slide into shoulder flexion and back.
26	Backstroke	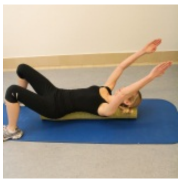	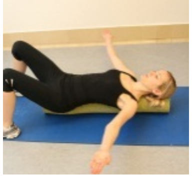	Supine on foam roller with legs bend and feet supported on the floor.<br>Move arms into flexion and abduction/ external rotation, action as if backstroke swimming.<br><br>Note: not subscapularis or infraspinatus tear.
27	Standing arm elevation in scapular plane	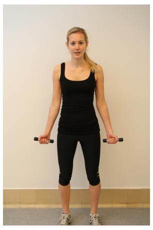	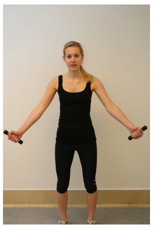	Stand with arms alongside the body. Forearms supinated. Perform shoulder abduction in the scapular plane.<br><br>Note: Can be performed on a wobble board and with light hand weights.

28	Standing shoulder flexion	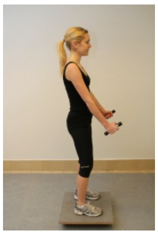	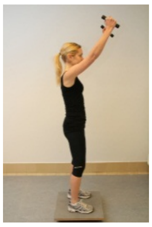	Stand with arms alongside the body. Forearms in neutral. Perform shoulder flexion.<br><br>Note: Can be performed on a wobble board and with light hand weights.
29	Sidelying external rotation	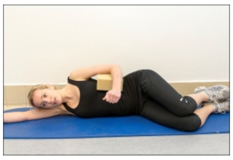	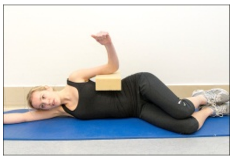	Side lying with a foam block between trunk and arm. Shoulder in neutral and elbow flexed 90 deg.<br>Perform external shoulder rotation against gravity.
30	Sidelying shoulder flexion	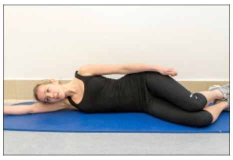	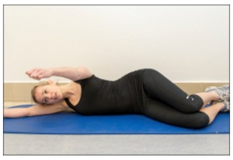	Side lying with arm alonside the body. Shoulder in neutral and elbow extended. Perform a shoulder flexion.
31	Prone drop/ grab ball	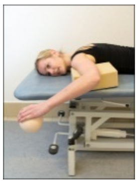		Prone on a bench. The arm is held in throwers position (shoulder abduction/ external rotation). Hold light weight ball. With a small ROM, alternately let the ball go and catch it again.
32	Prone external rotation w. 90 deg. elevation	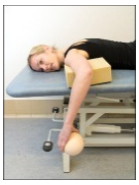	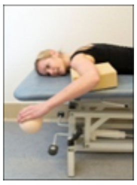	Prone on a bench with 90 deg. shoulder abduction and elbow flexion.<br>Hold light weight ball. Hand pointing to the floor.<br>External rotate to throwers position (shoulder abduction/ external rotation).<br>Can be performed without and with ball.
33	Standing protraction w/ pulley (throwing)	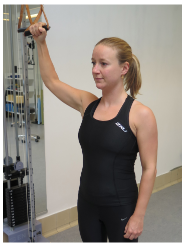	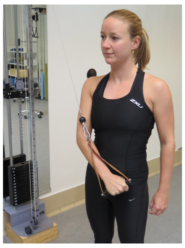	Standing with operated shoulder next to pulley. Stand up straight and lift the sternum. Shoulder in abduction/ external rotation. Simulating a throwing movement, pull towards contralateral hip.<br><br>Note: Can be performed with a long Theraband.
34	Standing retraction w/ pulley (throwing)	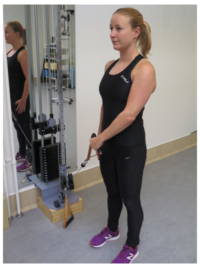	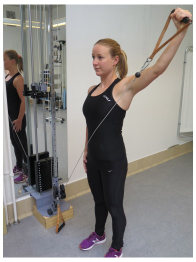	Standing with healthy shoulder next to pulley. Stand up straight and lift the sternum. Shoulder in adduction/ internal rotation. From contralateral hip, pull up and out into shoulder abduction/ external rotation.<br><br>Note: Can be performed with a long Theraband.

35	Supine external rotation (hand to neck)<br><br>(home program)	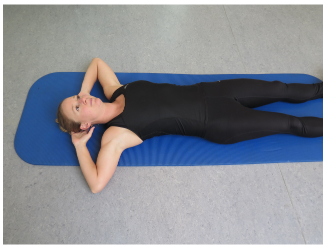	In supine slowly move the arms over the head into shoulder abduction/ external rotation. See if you can find rest in this position with the hands touching the back of the head.<br><br>Note: For less abduction/ external rotation, you may support the elbows with pillows.
36	Supine internal rotation (hand to lower back)<br><br>(home program)	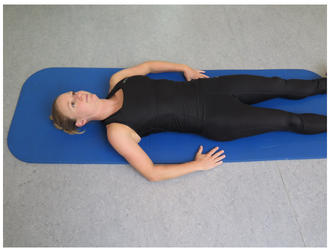	In supine slowly move the arms into shoulder abduction/ internal rotation. See if you can find rest in this position with the hands touching the matress or lower back.
Part 2 Isometrisk hold			
G	Squeeze ball	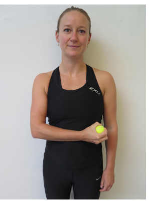	Stand up straight and lift the sternum. Squeeze a tennis ball. Maintain tension for 5 sec. and slowly relax.
H	Adduction	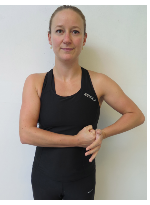	Stand up straight and lift the sternum. Hold arm in front of body with flexed elbow. Push fist against contralateral hand into adduction.
I	Internal rotation	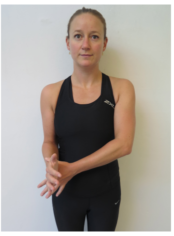	Stand up straight and lift the sternum. Forearm pointing forward with flexed elbow. Push hand against contralateral hand into internal rotation.<br><br>Note: Not subscapularis tear.
J	External rotation	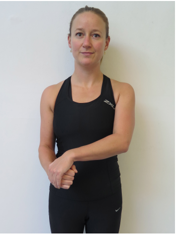 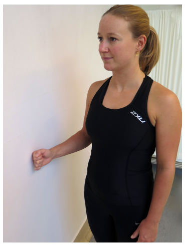	Stand up straight and lift the sternum. Forearm pointing forward with flexed elbow. Push hand against wall or contralateral hand into external rotation.<br><br>Note: Not infraspinatus tear.

K	Abduction	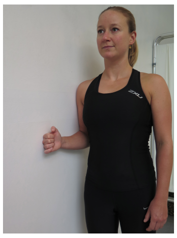	Stand up straight next to the wall and lift the sternum.<br>Forearm pointing forward with flexed elbow. Push forearm against wall into abduction.
L	Low row (against table)	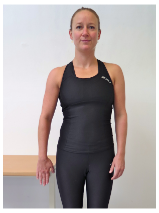	Stand up straight next to the table and lift the sternum.<br>The arm is alongside the body and hand against a table.<br>Push hand towards shoulder extension into table.
M	Flexion against wall	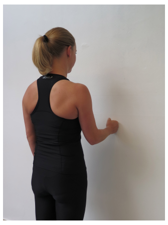	Stand up straight facing the wall and lift the sternum.<br>Forearm pointing forward with flexed elbow. Push fist against wall into shoulder flexion.
N	Extension against wall	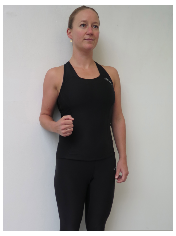	Stand up straight with back against wall and lift the sternum. Forearm pointing forward with flexed elbow. Push arm against wall into shoulder extension.
