# Supplementary material for: Progressive early passive and active exercise therapy after surgical rotator cuff repair – study protocol for a randomized controlled trial (the CUT-N-MOVE trial)
Source: Trials. 2018 Sep 3;19:470. doi: 10.1186/s13063-018-2839-5 (PMC6122575; doi:10.1186/s13063-018-2839-5)
Supplement: Supplementary file 2 — Exercise progression for the CUT-N-MOVE trial. (PDF 234 kb) [file 13063_2018_2839_MOESM2_ESM.pdf]

Rehabilitation program after rotator cuff surgery
Progressive

Bispebjerg Hospital — Herlev Hospital

In the clinic

NOTES

Sling week 1-2 postop:
Shoulder immobilized in standard fixed sling

Sling week 3-5 postop:
Shoulder immobilized in standard sling

Repetitions:

3 sets of 10 repetitions

Movement restrictions for AARAOM and AROM:
Flexion: 0°-90° (week 1-5)

Abduction: 0°-90° (week 1-5)

External rot.: 0° (week 1-2)

Internal rotation: no restrictions

Repaired tendon(s) are protected

Specify percieved pain on a scale from 0-10:

0 equals no pain
10 equals the worst imaginable

Pain below 5 is permitted during exercise therapy.

Pain should drop to below 2 after exercise therapy.

Part 1: Tendon protection

	Part 1: Tendon protection	Wk	1	2	3	4	5	6	7	8	9	10	12
A	Active elbow extension (supine/ standing)		•	•	•	•	•						
B	Vein pump (spread/collect fingers)		•	•	•	•	•						
C	Rotate forearm (supination/ pronation)		•	•	•	•	•						
D	Lift sternum (retraction of shoulders)		•	•	•	•	•						
E	Head lateral flexion		•	•	•	•	•						
F	Cervical flexion		•	•	•	•	•						

Exercises in sling

Part 2:

	Passive range of motion (by PT)	Wk	1	2	3	4	5	6	7	8	9	10	12
1	Flexion		•	•	•	•	•						
2	Abduction		•	•	•	•	•						
3	Supine external rotation w. 20-60 deg. abd.		•	•	•	•	•						
4	When needed manual guiding of scapula		•	•	•	•	•						
5	When needed manual therapy		•	•	•	•	•						

PROM

	Assisted active range of motion	Wk	1	2	3	4	5	6	7	8	9	10	12
6	Reach the foot (flexion)			•	•	•	•						
7	Wash the table (flexion on table)			•	•	•	•	•					
8	Supine bench press w/ broomstick			•	•	•	•	•	•				
9	Flexion/ scaption w/ jump rope			•	•	•	•	•	•				
10	Abduction w/ broomstick			•	•	•	•	•	•				
11	Supine external rotation w/ broomstick			•	•	•	•	•	•				

AAROM

	Isometric hold	Wk	1	2	3	4	5	6	7	8	9	10	12
G	Squeeze a ball			•	•	•	•	•	•	•			
H	Adduction			•	•	•	•	•	•	•			
I	Internal rotation (not subscapularis lesion)				•	•	•	•	•	•			
J	External rotation (not infraspinatus lesion)				•	•	•	•	•	•			
K	Abduction				•	•	•	•	•	•			
L	Low row (against table)				•	•	•	•	•	•			
M	Flexion against wall			•	•	•	•	•	•	•			
N	Extension against wall (flexed elbow)			•	•	•	•	•	•	•			

STRENGTH

	Active exercise closed and open chain	Wk	1	2	3	4	5	6	7	8	9	10	12
12	Downward pressure in abduction				•	•	•	•	•	•	•	•	•
13	Low row w/ Thera-Band (extension)				•	•	•	•	•	•	•	•	•
14	Incline plinth sliding (flexion)				•	•	•	•	•	•	•	•	•
15	Sitting bilateral downward press				•	•	•	•	•	•	•	•	•
16	High row with pulley					•	•	•	•	•	•	•	•
17	Fitter					•	•	•	•	•	•	•	•
18	Prone low row					•	•	•	•	•	•	•	•
19	Supine scaption on sloping board					•	•	•	•	•	•	•	•
20	Supine internal rotation w/ pulley					•	•	•	•	•	•	•	•
21	Supine external rotation w/ pulley					•	•	•	•	•	•	•	•
22	Standing internal rotation w/ pulley						•	•	•	•	•	•	•
23	Lawn mover						•	•	•	•	•	•	•
24	Standing external rotation w/ pulley						•	•	•	•	•	•	•
25	Wall slide							•	•	•	•	•	•
26	Backstroke							•	•	•	•	•	•
27	Standing arm elevation in scapular plane							•	•	•	•	•	•
28	Standing shoulder flexion							•	•	•	•	•	•
29	Sidelying external rotation							•	•	•	•	•	•
30	Sidelying shoulder flexion							•	•	•	•	•	•
31	Prone drop/ grab ball											•	•
32	Prone external rotation w. 90 deg. elevation											•	•
33	Standing protraction w/ pulley (throwing)											•	•
34	Standing retraction w/ pulley (throwing)											•	•

AROM

Level 1 (12–21) — **Level 2** (22–28) — **Level 3** (29–34)

By Birgitte Hougs Kjær, Copenhagen University Hospital Bispebjerg Frederiksberg 2017

Rehabilitation program after rotator cuff surgery Usual care

Bispebjerg Hospital — Herlev Hospital

In the clinic

NOTES

Sling week 1-2 postop: Shoulder immobilized in standard fixed sling

Sling week 3-5 postop: Shoulder immobilized in standard sling

Repetitions:

3 sets of 10 repetitions

Movement restrictions:

No active movement within the first 5 weeks postop.

Specify percieved pain on a scale from 0-10:

0 equals no pain 10 equals the worst imaginable

Pain below 5 is permitted during exercise therapy.

Pain should drop to below 2 after exercise therapy.

		Wk	1	2	3	4	5	6	7	8	9	10	12
**Part 1:**	**Tendon protection**	Wk	1	2	3	4	5	6	7	8	9	10	12
**A**	Active elbow extension (supine/ standing)		•	•	•	•	•						
**B**	Vein pump (spread/collect fingers)		•	•	•	•	•						
**C**	Rotate forearm (supination/ pronation)		•	•	•	•	•			Exercises in sling			
**D**	Lift sternum (retraction of shoulders)		•	•	•	•	•						
**E**	Head lateral flexion		•	•	•	•	•						
**F**	Cervical flexion		•	•	•	•	•						
**Part 2:**													
	**Passive range of motion (by PT)**	Wk	1	2	3	4	5	6	7	8	9	10	12
**1**	Flexion		•	•	•	•	•						
**2**	Abduction		•	•	•	•	•						
**3**	Supine external rotation w. 20-60 deg. abd.		•	•	•	•	•			PROM			
**4**	When needed manual guiding of scapula		•	•	•	•	•						
**5**	When needed manual therapy		•	•	•	•	•						
	**Assisted active range of motion**	Wk	1	2	3	4	5	6	7	8	9	10	12
**6**	Reach the foot (flexion)					•	•	•	•	•	•		
**7**	Wash the table (flexion on table)							•	•	•	•	•	
**8**	Supine bench press w/ broomstick	AAROM						•	•	•	•	•	•
**9**	Flexion/ scaption w/ jump rope							•	•	•	•	•	•
**10**	Abduction w/ broomstick							•	•	•	•	•	•
**11**	Supine external rotation w/ broomstick							•	•	•	•	•	•
	**Active exercise closed and open chain**	Wk	1	2	3	4	5	6	7	8	9	10	12
**12**	Downward pressure in abduction							•	•	•	•	•	•
**13**	Low row w/ Thera-Band (extension)			AROM				•	•	•	•	•	•
**14**	Incline plinth sliding (flexion)							•	•	•	•	•	•
**15**	Sitting bilateral downward press							•	•	•	•	•	•
**16**	High row with pulley								•	•	•	•	•
**17**	Fitter	**Level 1**							•	•	•	•	•
**18**	Prone low row								•	•	•	•	•
**19**	Supine scaption on sloping board								•	•	•	•	•
**20**	Supine internal rotation w/ pulley								•	•	•	•	•
**21**	Supine external rotation w/ pulley								•	•	•	•	•
**22**	Standing internal rotation w/ pulley								•	•	•	•	•
**23**	Lawn mover								•	•	•	•	•
**24**	Standing external rotation w/ pulley								•	•	•	•	•
**25**	Wall slide								•	•	•	•	•
**26**	Backstroke	**Level 2**							•	•	•	•	•
**27**	Standing arm elevation in scapular plane								•	•	•	•	•
**28**	Standing shoulder flexion								•	•	•	•	•
**29**	Sidelying external rotation								•	•	•	•	•
**30**	Sidelying shoulder flexion								•	•	•	•	•
**31**	Prone drop/ grab ball											•	•
**32**	Prone external rotation w. 90 deg. elevation			**Level 3**								•	•
**33**	Standing protraction w/ pulley (throwing)											•	•
**34**	Standing retraction w/ pulley (throwing)											•	•

By Birgitte Hougs Kjær, Copenhagen University Hospital Bispebjerg Frederiksberg 2017

Rehabilitation program after rotator cuff surgery Progressive

At home

NOTES

Sling week 1-2 postop:
Shoulder immobilized in standard fixed sling

Sling week 3-5 postop:
Shoulder immobilized in standard sling

Repetitions:
3 sets of 10 repetitions

Frequency:
Range of motion exercises is performed daily.

Strength exercises is performed on the days without PT training.

From week 6 both closed and open chain exercises are performed.

Specify percieved pain on a scale from 0-10:

0 equals no pain
10 equals the worst imaginable

Pain below 5 is permitted during exercise therapy.

Pain should drop to below 2 after exercise therapy.

**Part 1:**	**Tendon protection**	Wk	1	2	3	4	5	6	7	8	9	10	12
**A**	Active elbow extension (supine/ standing)		•	•	•	•	•						
**B**	Vein pump (spread/collect fingers)		•	•	•	•	•						
**C**	Rotate forearm (supination/ pronation)		•	•	•	•	•						
**D**	Lift sternum (retraction of shoulders)		•	•	•	•	•						
**E**	Head lateral flexion		•	•	•	•	•						
**F**	Cervical flexion		•	•	•	•	•						

Exercises in sling

Part 2:

	**Assisted active range of motion**	Wk	1	2	3	4	5	6	7	8	9	10	12
**6**	Reach the foot (flexion)			•	•	•	•						
**7**	Wash the table (flexion on table)			•	•	•	•	•					
**8**	Supine bench press w/ broomstick			•	•	•	•	•	•				
**10**	Abduction w/ broomstick			•	•	•	•	•	•				
**11**	Supine external rotation w/ broomstick			•	•	•	•	•	•				

ASSISTED ACTIVE

Performed without sling

	**Isometric hold**	Wk	1	2	3	4	5	6	7	8	9	10	12
**M**	Flexion against wall			•	•	•	•	•					
**N**	Extension against wall (flexed elbow)			•	•	•	•	•					
**I**	Internal rotation (not subscapularis lesion)					•	•	•	•	•			
**J**	External rotation (not infraspinatus lesion)					•	•	•	•	•			
**K**	Abduction						•	•	•	•			

STRENGTH

Performed without sling

	**Active range of motion**	Wk	1	2	3	4	5	6	7	8	9	10	12
**13**	Low row w/ Thera-Band (extension)							•	•	•	•	•	•
**18**	Prone low row							•	•	•	•	•	•
**35**	Supine external rotation (hand to neck)							•	•	•	•	•	•
**36**	Supine internal rotation (hand to lower back)							•	•	•	•	•	•

ACTIVE

By Birgitte Hougs Kjær, Copenhagen University Hospital Bispebjerg Frederiksberg 2017

Rehabilitation program after rotator cuff surgery Usual care

Bispebjerg Hospital — Herlev Hospital

At home

NOTES

Sling week 1-2 postop:
Shoulder immobilized in standard fixed sling

Sling week 3-5 postop:
Shoulder immobilized in standard sling

Repetitions:
3 sets of 10 repetitions

Frequency:
Range of motion exercises is performed daily.

Strength exercises is performed on the days without PT training.

From week 6 both closed and open chain exercises are performed.

Specify percieved pain on a scale from 0-10:

0 equals no pain
10 equals the worst imaginable

Pain below 5 is permitted during exercise therapy.

Pain should drop to below 2 after exercise therapy.

**Part 1:**	**Tendon protection**	Wk	1	2	3	4	5	6	7	8	9	10	12
**A**	Active elbow extension (supine/ standing)		•	•	•	•	•						
**B**	Vein pump (spread/collect fingers)		•	•	•	•	•						
**C**	Rotate forearm (supination/ pronation)		•	•	•	•	•						
**D**	Lift sternum (retraction of shoulders)		•	•	•	•	•						
**E**	Head lateral flexion		•	•	•	•	•						
**F**	Cervical flexion		•	•	•	•	•						

Exercises in sling

Part 2:

	**Assisted active range of motion**	Wk	1	2	3	4	5	6	7	8	9	10	12
**6**	Reach the foot (flexion)					•	•	•	•	•	•		
**7**	Wash the table (flexion on table)							•	•	•	•	•	
**8**	Supine bench press w/ broomstick							•	•	•	•	•	•
**10**	Abduction w/ broomstick							•	•	•	•	•	•
**11**	Supine external rotation w/ broomstick							•	•	•	•	•	•

ASSISTED ACTIVE

	**Active range of motion**	Wk	1	2	3	4	5	6	7	8	9	10	12
**13**	Low row w/ Thera-Band (extension)							•	•	•	•	•	•
**18**	Prone low row							•	•	•	•	•	•
**35**	Supine external rotation (hand to neck)							•	•	•	•	•	•
**36**	Supine internal rotation (hand to lower back)							•	•	•	•	•	•

ACTIVE

By Birgitte Hougs Kjær, Copenhagen University Hospital Bispebjerg Frederiksberg 2017
